# Tetracyclic Endiandric Acids Isolated from *Endiandra kingiana* Gamble (Lauraceae) with *In vitro* and Molecular Docking Studies of *β*-Glucuronidase Activity

**DOI:** 10.21315/tlsr2026.37.3.15

**Published:** 2026-10-15

**Authors:** Nurul Najihah Husna Tanazi, Ahmad Nazif Aziz, M. Iqbal Choudhary, Sadia Siddiq, Muhamad Aqmal Othman, Mohammad Tasyriq Che Omar, Cécile Apel, Mohamad Nurul Azmi

**Affiliations:** 1 Natural Products and Synthesis Organic Research Laboratory (NPSO), School of Chemical Sciences, School of Chemical Sciences, Universiti Sains Malaysia, 11800 USM Pulau Pinang, Malaysia; 2 Faculty of Science and Marine Environment, Universiti Malaysia Terengganu, 21030, Terengganu, Malaysia; 3 H.E.J Research Intitute of Chemistry, International Centre for Chemical and BIological Sciences, University of Karachi, Karachi-75270, Pakistan; 4 Department of Chemistry, Faculty of Science, University of Malaya, 50603, Kuala Lumpur, Malaysia; 5 Biological Section, School of Distance Education, Universiti Sains Malaysia, 11800 USM, Pulau Pinang, Malaysia; 6 Institute de Chimie des Substances Naturelles, CNRS, UPR 2301, Université Paris-Saclay, 91108 Gif-Sur-Yvette, France

**Keywords:** Endiandric Acid Derivatives, *Endiandra kingiana*, *β*-Glucuronidase Inhibitor, Molecular Docking, Asid Endiandric Derivatif, Endiandra kingiana, Perencat β-Glucuronidase, Dok Molekul

## Abstract

*β*-glucuronidase is a lysosomal enzyme that hydrolyses of glucuronide conjugates, playing a significant role in drug and xenobiotic metabolism as well as various disease processes. Elevated activity of this enzyme reactivates glucuronidated carcinogens, promoting tumorigenesis and inflammation. Thus, inhibiting *β*-glucuronidase has emerged as a promising therapeutic strategy, particularly for the prevention of colonic carcinogenesis. In this study, three endiandric acid derivatives named kingianic acid A (**1**), tsangibeilin B (**2**), and endiandric acid M (**3**) that isolated from *Endiandra kingiana* bark, were evaluated for their *β*-glucuronidase inhibitory activity. Their chemical structures were identified using spectroscopic data and by comparison with published reports. *In vitro* enzyme inhibition assays revealed that compound **2** (IC_50_ value of 7.5 ± 2.77 μM) exhibited the most potent inhibition compared to compound **1** (IC_50_ value of 292 ± 5.40 μM) and the positive control, *D*-saccharic acid-1,4-lactone (IC_50_ value of 45.75 ± 2.16 μM). In addition, molecular docking studies further supported these findings, demonstrating that compound **2** exhibited stronger binding affinity to *β*-glucuronidase (−9.9 ± 0.1 kcal/mol) than compound **1** (IC_50_ value of 292 ± 5.40 μM) and *D*-saccharic acid-1,4-lactone (−7.0 ± 0.1 kcal/mol). These results highlight the potential of compound **2** as a lead scaffold for the development of *β*-glucuronidase inhibitors and contribute to the growing interest in plant-derived compounds for therapeutic applications.

## Abstrak

β-glucuronidase ialah enzim lisosom yang menghidrolisis konjugat glukuronida, memainkan peranan penting dalam metabolisme ubat dan xenobiotik serta pelbagai proses penyakit. Aktiviti enzim ini yang tinggi boleh mengaktifkan semula karsinogen yang diglukuronidkan, seterusnya menggalakkan tumorigenesis dan keradangan. Oleh itu, menghalang β-glucuronidase muncul sebagai strategi terapeutik yang menjanjikan, terutamanya untuk pencegahan karsinogenesis kolon. Dalam kajian ini, tiga derivatif asid endiandrik yang dinamakan asid kingianik A (**1**), tsangibeilin B (**2**), dan asid endiandrik M (**3**) yang diasingkan daripada kulit pokok Endiandra kingiana, telah dinilai untuk aktiviti perencatan β-glucuronidase mereka. Struktur kimia mereka dikenalpasti menggunakan data spektroskopi dan dengan membandingkan dengan laporan yang diterbitkan. Ujian penghambatan enzim in vitro menunjukkan bahawa sebatian **2** (nilai IC50 7.5 ± 2.77 μM) menunjukkan penghambatan paling kuat berbanding sebatian **1** (nilai IC_50_ 292 ± 5.40 μM) dan kawalan positif, D-asid sakarik-1,4-lakton (nilai IC_50_ 45.75 ± 2.16 μM). Selain itu, kajian docking molekul turut menyokong penemuan ini, menunjukkan bahawa sebatian **2** mempunyai afiniti pengikatan lebih kuat kepada β-glukuronidase (−9.9 ± 0.1 kcal/mol) berbanding sebatian **1** (nilai IC50 292 ± 5.40 μM) dan D-asid sakarik-1,4-lakton (−7.0± 0.1 kcal/mol). Keputusan ini menekankan potensi sebatian **2** sebagai rangka utama untuk pembangunan penghalang β-glukuronidase dan menyumbang kepada minat yang semakin meningkat terhadap sebatian berasaskan tumbuhan untuk aplikasi terapeutik.

**HIGHLIGHTS**

- The isolation and purification from Malaysian *Endiandra kingiana* Gamble (Lauraceae) bark led to the isolation of three endiandric acids.
- The *in vitro β*-glucuronidase activity showed that tsangibeilin B and kingianic acid A exhibited good activity with IC_50_ value of 7.5 μM and 292 μM, respectively.
- Tsangibeilin B has a great potential for drug development against *β*-glucuronidase since it showed stronger inhibitory activity in *in vitro* assay and binding affinity in molecular docking study.

## INTRODUCTION

*β*-glucuronidase is a lysosomal glycosidase enzyme found in many organisms, including bacteria and humans, that hydrolyses *β*-D-glucuronide bonds. By cleaving glucuronide conjugates, it can release active aglycones from their glucuronate (detoxified) forms (Phong *et al*. 2025; Rauf *et al*. 2025). This enzyme has the capability of hydrolysing conjugated compounds, including glucuronic acid, into their derivatives and free glucuronic acid. By hydrolysing glucuronide conjugates into free aglycones and glucuronic acid, *β*-glucuronidase can paradoxically aid in detoxification or cause toxin reactivation. While glucuronidation is a key phase II detoxification pathway, *β*-glucuronidase can reverse this process, potentially leading to reabsorption of toxins (Phong *et al*. 2022). In humans, *β*-glucuronidase can be detected in many organs and fluids, including the serum, kidney, liver, lung, and the digestive system (Ullah *et al*. 2021).

Dysregulation of *β*-glucuronidase activity has been associated with a variety of pathological conditions, including cancer, HIV, neuropathy, rheumatoid arthritis, diabetes mellitus and microbial infections (Rauf *et al*. 2025; Kamel *et al*. 2024; Awolade *et al*. 2020). Additionally, *β*-glucuronidase has been reported to be released into synovial fluid under inflammatory conditions, such as rheumatoid arthritis (Rahim *et al*. 2024; Taha *et al*. 2018). Elevated *β*-glucuronidase activity plays a critical role in carcinogenesis by reactivating glucuronide-conjugated carcinogens, thereby promoting tumour progression and potentially contributing to chemotherapy resistance (Alwaili *et al*. 2025). Recent findings indicate that *β*-glucuronidase is a key role tumour progression by promoting cell proliferation, invasion, and metastasis (Tranoy-Opalinski *et al*. 2014). The involvement of *β*-glucuronidase in colon cancer is supported by evidence that higher intestinal levels of the enzyme correlate with a higher incidence of colon carcinoma (Taha *et al*. 2016). Thus, the discovery and development of *β*-glucuronidase inhibitors represent an effective strategy for preventing and treating various diseases.

Natural products and synthetic scaffolds have shown considerable potential as *β*-glucuronidase inhibitors, with several progressing toward clinical application despite moderate to weak pharmacokinetic profiles. Plants, in particular, have been recognised as a prolific source of bioactive compounds and have been extensively studied for their medicinal properties. In recent years, these natural compounds have attracted renewed interest as promising scaffolds for the development of modern therapeutics, a trend that has been well-documented (Rauf *et al*. 2025; Dzouemo *et al*. 2022). *Endiandra kingiana* Gamble (*E. kingiana*), a member of the Lauraceae (laurel) family, has attracted significant scientific interest due to its traditional medicinal use (Lenta *et al*. 2015). Over the past decades, species within the *Endiandra* genus have been extensively studied, particularly for their production of unique polyketide compounds, such as endiandric acids and kingianins (Azmi *et al*. 2014; 2016). Several compounds previously isolated from *E. kingiana* have demonstrated bioactivity against various targets, including anti-apoptotic proteins (Bcl-xL and Mcl-1) (Leverrier *et al*. 2011; Azmi *et al*. 2014; 2016), dengue virus type 2 NS2B/NS3 serine protease (Sulaiman *et al*. 2019), and models related to diabetes mellitus (Abu Bakar *et al*. 2020; Azmi *et al*. 2021; Tanazi *et al*. 2023).

In our previous work, ten compounds were successfully isolated from *E. kingiana* bark and their structures were identified, including three endiandric acid compounds: kingianic acid A (**1**), tsangibeilin B (**2**) and endiandric acid M (**3**) (Tanazi *et al*. 2023). In our continuous effort to evaluate the biological properties of medicinal plants, endiandric acid derivatives were tested in a *β*-glucuronidase inhibition assay. In addition, molecular docking studies were conducted to further investigate the mechanism of inhibition, providing insights into the therapeutic potential of these compounds.

## MATERIALS AND METHOD

### Experimental Procedures

The 1D and 2D NMR spectra were recorded in deuterated chloroform (CDCl_3_; Merck, deuteration degree min. 99.8%) using a Bruker Avance 400 MHz NMR spectrometer. Data were analyses via TopSpin software package. CDCl_3_ was used as the solvent, and chemical shifts were referenced internally to the solvent signals (^1^H δ_H_ 7.24; ^13^C δ_C_ 77.23). The mass spectra were obtained on a Thermo Scientific LCMS-Orbitrap system. The UV absorption spectra were recorded using a Shimadzu UV-1900i UV-Visible Spectrophotometer with methanol (CH_3_OH) as the solvent. IR spectra were recorded on a Bruker INVENIO S FT-IR spectrometer with CHCl_3_ as the solvent. RHPLC was performed using an LC-9130 preparative system (JAI Co. Ltd., Tokyo, Japan) equipped with a JAIGEL-ODS-AP-30 preparative column (15 μm, 30 mm × 250 mm) and multi-wavelength UV detector. Silica gel 60 (Merck, 0.040 mm–0.063 mm) was used for column chromatography (CC). Aluminium-supported silica gel 60 F_254_ plates 20 cm × 20 cm were used for thin layer chromatography (TLC) (Merck, Germany). TLC spots were visualised under UV light (254 nm and 365 nm) followed by spraying with anisaldehyde reagent and heating with a heat gun. TLC was used to monitor fractionation during purification and to assess compound purity, while structural elucidation was performed using spectroscopic methods.

#### Chemical and reagents

All solvents, except those used for purification were of analytical reagent (AR) grade. HPLC grade solvents used for RHPLC separation were supplied by Merck (Germany) and Fisher Scientific (USA). The *E. coli β*-glucuronidase enzyme (EC 3.2.1.31, G7396) was purchased from Sigma-Aldrich (St. Louis, MO, USA).

#### Plant material

The bark of *Endiandra kingiana* Gamble was collected in 2006 from the Reserved Forest of Sg. Temau, Kuala Lipis, Pahang, on the East Coast of Peninsular Malaysia. The species was identified by Teo L. E, a botanist from the Faculty of Science, Universiti Malaya. A voucher specimen (KL5243) was deposited in the Herbarium of the Department of Chemistry, Faculty of Science, Universiti Malaya, Kuala Lumpur, Malaysia.

#### Extraction, isolation and characterisation of compounds 1, 2 and 3

The compounds were isolated and characterised according to previously described methods (Tanazi *et al*. 2023). Three endiandric acids were successfully isolated using the RHPLC system from *n*-hexane-soluble and methanol-soluble subfractions. Kingianic acid A (**1**), tsangibeilin B (**2**) and endiandric acid M (**3**) were separated on an ODS column using acetonitrile-water (80:20, v/v) as the mobile phase under isocratic conditions at a flow rate of 10.0 mL/min, with detection at 210, 254, 270 and 360 nm.

Kingianic Acid (**1**): Colourless oil. Yield: 12.0 mg. HR-ESI-MS *m/z*: 325.1430 [M+H]^+^ (calcd. 325.3798), C_20_H_21_O_4_; UV (CH_3_OH) **λ**_max_ nm: 289, 233; FTIR **ν**_maz_ cm^−1^: 3352 (O-H acid), 1708 (C=O acid), 1037 and 921 (methylenedioxy group); ^1^H NMR (400 MHz, CDCl_3_) **δ**_H_: 1.54 (d, J = 12.8 Hz, H 6α), 1.73 (m, H-3), 1.88 (m, H-6β), 1.96 (t, J = 8.0 Hz, H-4), 2.31 (br t, J = 6.2 Hz, H-5), 2.40 (m, H-2), 2.53 (t, J = 4.9 Hz, H-7), 2.70 (m, H-1), 2.73 (m, H-1α’), 2.78 (m, H-1β’), 2.84 (d, J = 3.6 Hz, H-8), 2.96 (m, H-9), 5.90 (s, H-8’), 6.19 (br t, J = 3.8 Hz, H-10), 6.19 (br t, J = 3.8 Hz, H-11), 6.59 (d, J = 7.9 Hz, H-7’), 6.63 (s, H-3’), 6.72 (d, J = 7.0 Hz, H-6’); ^13^C NMR (100 MHz, CDCl_3_) **δ**_C_: 34.8 (C-9), 38.2 (C-7), 38.4 (C-6), 38.8 (C-3), 39.7 (C-2), 39.8 (C-5), 40.5 (C-4), 41.7 (C-1’), 41.8 (C-1), 48.9 (C-8), 100.8 (C-8’), 108.1 (C-6’), 109.1 (C-3’), 121.7 (C-7’), 131.3 (C-10), 132.0 (C-11), 134.7 (C-2’), 145.6 (C-5’), 147.5 (C-4’), 180.3 (C=O).

Tsangibeilin B (**2**): Colourless oil. Yield: 5.2 mg. HR-ESI-MS *m/z*: 373.3505 [M+Na]^+^ (calcd. 373.3996), C_22_H_22_O_4_Na; UV (CH_3_OH) **λ**_max_ nm: 286, 235; FTIR **ν**_max_ cm^−1^: 3401 (O-H acid), 1698 (C=O acid), 1039 and 931 (methylenedioxy group); ^1^H NMR (400 MHz, CDCl_3_) **δ**_H_: 1.32 (t, J = 7.4 Hz, H-2α), 1.53 (dd, J = 11.7 Hz, 5.6 Hz, H-2β), 1.74 (m, H-13), 1.78 (m, H-11), 2.38 (m, H-1), 2.41 (m, H-10), 2.53 (m, H-3), 2.67 (br d, J = 8.0 Hz, 8.2 Hz, H-12), 2.69 (m, H-1α’), 2.71 (m, H-1β’), 2.98 (m, H-7), 3.00 (m, H-6), 5.41 (m, H-8), 5.43 (m, H-9), 5.72 (dt, J = 9.7 Hz, 2.9 Hz, H-5), 5.91 (s, H-8’), 6.19 (dt, J = 9.7 Hz, 2.3 Hz, H-4), 6.6 (dd, J = 7.7, 1.4 Hz Hz, H-7’), 6.63 (d, J = 1.4 Hz, H-3’), 6.70 (d, J = 7.9 Hz, H-6’); ^13^C NMR (100 MHz, CDCl_3_) **δ**_C_: 32.9 (C-7), 32.9 (C-12), 34.4 (C-1), 34.7 (C-2), 36.9 (C-3), 40.9 (C-10), 42.0 (C-13), 42.6 (C-1’), 47.1 (C-11), 49.0 (C-6), 100.7 (C-8’), 108.0 (C-6’), 109. (C-3’), 121.4 (C-7’), 123.9 (C-5), 129.1 (C-8), 129.8 (C-9),134.4 (C-4), 134.5 (C-2’), 145.6 (C-5’), 147.5 (C-4’), 178.3 (C=O).

Endiandric acid M (**3**): Yellowish oil. Yield: 1.9 mg. HR-ESI-MS *m/z*: 351.1586 [M+H]^+^ (calcd. 351.4174), C_22_H_23_O_4_; UV (CH_3_OH) **λ**_max_ nm: 287, 232; FTIR **ν**_max_ cm-1: 3401 (O-H acid), 1708 (C=O acid), 1040 and 933 (methylenedioxy group); ^1^H NMR (400 MHz, CDCl_3_) **δ**_H_: 1.58 (d, J = 12.7 Hz, H-6α), 1.70 (m, H-3), 1.84 (m, H-6β), 1.90 (t, J = 4.8 Hz, H-4), 2.08 (t, J = 8.2 Hz, H-7), 2.32 (br t, J = 7.1 Hz, H-5), 2.42 (m, H-2), 2.51 (m, H-9), 2.72 (m, H-1), 2.73 (m, H-1α’), 2.75 (m, H-1β’), 2.78 (m, H-8), 5.69 (d, J = 15.7 Hz, H-13), 5.9 (s, H-8’), 6.12 (br t, J = 7.4 Hz, H-11), 6.14 (t, J = 7.4 Hz, H-10), 6.60 (d, J = 7.9 Hz, H-7’), 6.64 (s, H-3’), 6.70 (d, J = 7.9 Hz, H-6’), 6.84 (dd, J = 15.7 Hz, 8.2 Hz, H-12); ^13^C NMR (100 MHz, CDCl_3_) **δ**_C_: 37.2 (C-9), 38.8 (C-6), 39.6 (C-3), 39.5 (C-5), 39.9 (C-2), 40.0 (C-7), 41.7 (C-4), 41.8 (C-1’), 42.2 (C-1), 47.3 (C-8), 100.7 (C-8’), 108.1 (C-6’), 109.0 (C-3’), 118.4 (C-13), 121.5 (C-7’), 130.7 (C-11), 132.5 (C-10), 134.8 (C-2’), 145.7 (C-5’), 147.6 (C-4’), 156.7 (C-12), 171.2 (C=O).

## *β*-GLUCURONIDASE INHIBITORY

The *β*-glucuronidase inhibition assay was conducted according to the standard method (Liew *et al*. 2020) with significant modifications. In a 96-well microplate, the reaction mixture containing 185 μL of acetate buffer (0.1 M, pH 7), 5 μL of test compound solution, and 10 μL of enzyme (1U) was added. The mixture was incubated for 30 min at 37°C. Subsequently, 50 μL of *p*-nitrophenyl-*β*-D-glucuronide was added, and the readings were recorded. Enzyme inhibitory activity was assessed by measuring absorbance at 405 nm using a SpectraMax multiplate reader (Molecular Devices, CA, USA), following the conversion of the substrate, *p*-nitrophenyl-*β*-D-glucuronide. Test compounds were analysed in triplicate. *D*-Saccharic acid 1,4-lactone was used as a positive control. The IC_50_ values were determined by serial dilution and calculated using EZ-Fit enzyme kinetics software (Perrella Scientific, Amherst, NH, USA).

### Molecular docking

The crystallographic structure of *E. coli β*-glucuronidase enzyme with inhibitor was obtained from the Protein Data Bank (https://www.rcsb.org/), with PDB ID of 6LEL. Before docking, the crystal PDB file was processed using UCSF Chimera version 1.14 (University of California, CA, USA). Water molecules and unrelated heteroatoms (i.e., any ion molecules and atoms not belonging to protein) were removed. The receptor and inhibitor were separated from the complex into individual structures, which were then minimised using steepest descent steps. Polar hydrogens and Gasteiger charges were added (Wang *et al*. 2006). The ligands (compounds **1**–**3**) were drawn using ChemDraw Professional 22.00 software (PerkinElmer Informatics, Massachusetts, USA) and converted to three-dimensional (3D) structures with Chem3D. Geometry optimisation and energy minimisation were performed using the MM2 force field. The final structures were saved in .pdb format.

To identify the binding site in the *β*-glucuronidase protein, the grid size was set to 16, 16 and 16 along with the X-. Y-, and Z-axes with a 0.375Å grid spacing, respectively. The grid centre along the X-, Y- and Z-axes was set to −23.14 Å, 9.146 Å and −3.011 Å, respectively. Molecular docking was performed using AutoDock Vina (Trott & Olson 2010; Eberhardt *et al*. 2021) with three independent docking runs per ligand. Docking experiments were conducted for compound **1**, compound **2** and the standard inhibitor, *D*-saccharic acid 1,4-lactone. The 2D diagram of receptor-ligand interactions were visualised with Biovia Discovery Studio Visualizer Client 2020 (Dassault Systèmes, California, USA).

### Statistical Analysis

All values are expressed as mean ± standard deviation (SD, *n* = 3). The IC_50_ values were calculated by probit analysis using the Statistical Package for the Social Sciences (SPSS, IBM Corp., Chicago, USA). A lower IC_50_ value indicates stronger *β*-glucuronidase inhibitory activity.

## RESULTS AND DISCUSSSION

### Structural Characterisation of Isolated Compounds

Three compounds, **1**–**3** were isolated from the *n*-hexane and methanol-soluble fractions. Their structures (Fig. 1) were determined spectroscopically by 1D-NMR, 2D-NMR, UV, IR and LC-MS, as well as by comparison with literature data (Azmi *et al*. 2014). The structure determination of these compounds is described in the present paper (Tanazi *et al*. 2023). The three isolated compounds are identified as kingianic acid A (**1**), tsangibeilin B (**2**) and endiandric acid M (**3**).

### *β*-Glucuronidase Inhibitory Activity of Isolated Compounds

In this *in vitro* assay, compounds **1** and **2** were evaluated for their ability to inhibit *β*-glucuronidase, using *D*-saccharic acid 1,4-lactone (DSA), a well-known inhibitor of *β*-glucuronidase, as the positive control (Karunairatnam & Levvy 1949). Compound **3** was not tested due to insufficient quantity. The results displayed in Table 1, showed that compound **2** exhibited strong inhibition of *β*-glucuronidase activity with an IC_50_ value of 7.5 ± 2.77 μM, which was lower than that of the positive control, DSA (45.75 ± 2.16 μM). This indicates that compound **2** has more potent inhibitory effects (Phong *et al*. 2025). In contrast, compound **1** weakly inhibited *β*-glucuronidase, with an IC_50_ of 292 ± 5.40 μM. Table 1 shows the IC_50_ values of the isolated compounds against *β*-glucuronidase.

### Molecular Docking Analysis

According to the enzyme inhibition results (Table 1), compound **2** was identified as a potent inhibitor of the *β*-glucuronidase enzyme. To further understand the binding interaction between compound **2** and the *β*-glucuronidase enzyme (PDB ID: 6LEL), *in silico* molecular docking studies were conducted. Initially, to assess the efficacy of docking, re-docking was performed using the co-crystallised ligands. The re-docked co-crystallised (purple) was then superimposed on the native co-crystallised (blue) ligand as shown in Fig. 2, and the root mean square deviation (RMSD) was calculated. Since RMSD < 1.5 Å (Taha *et al*. 2016), the docking method and receptor model (PDB: 6LEL) were considered valid.

The docking analysis revealed that compound **2** exhibited potent binding affinity to *β*-glucuronidase with a binding energy of −9.9 ± 0.1 kcal/mol, compared to compound **1** (−9.3 ± 0.1 kcal/mol) and the standard inhibitor (−7.0 ± 0.1 kcal/mol) (Table 2). These results indicate that compound **2** forms more favourable interactions within the enzyme’s active site, as further supported by its 3D binding orientations (Figure 3) and molecular interactions (Table 3).

Compound **2** exhibits strong binding affinity due to the formation of hydrogen bond between its carboxylic acid group and the active site residue, Asp163 at a distance of 3.14 Å. Additionally, the methylenedioxyphenyl group of compound **2** demonstrates stabilising hydrophobic interactions, including π–alkyl, π–sigma, and π–π stacked interactions with Met447, Tyr472 and Leu361. These interactions enhance ligand–protein complementarity in the active site. The presence of hydrogen bonds and hydrophobic interactions collectively contributes to the stability of the ligand-protein complex (Munawaroh *et al*. 2020; Shanak *et al*. 2021). Moreover, Arg562, Glu413, Trp549, His330, Phe164, Val355, His162, Asn412, Phe161, Val446 and Phe448 residues form van der Waals interactions with compound **2**. In addition to hydrogen bonding and π–π stacking interactions, van der Waals forces contribute significantly to the stabilisation of protein–ligand complexes, especially when these interactions involve residues within the catalytic pocket (Ferreira *et al*. 2015; Tsai *et al*. 2001). Compound **2** forms multiple van der Waals contacts with surrounding amino acid residues, including a notable interaction with Glu413, a known catalytic residue in *β*-glucuronidase.

The binding mode orientation of compound **1** shows the formation of hydrogen bond between the carboxylic acid moiety with Glu504, Asn566 and Lys568, as well as between the methylenedioxyphenyl group and Tyr472. Additionally, the methylenedioxyphenyl moiety forms π-π stacking with Tyr472, further enhancing the aromatic interaction. Furthermore, the amino acid residue Leu361 forms alkyl and π-alkyl interactions with cyclohexane and methylenedioxyphenyl group, respectively. The other part of the compound forms van der Waals interactions with Glu413, Arg562, Tyr468, Asp163, Trp549, Met447 and Val473. Fig. 3 illustrates the binding interactions of compounds **1**, **2**, and *D*-saccharic acid 1,4-lactone with *β*-glucuronidase protein.

The 3D visualisation of the docking poses shows that compound **2** achieves a deeply embedded orientation within the catalytic pocket of *β*-glucuronidase protein. It is tightly positioned in the active site cleft, forming multiple van der Waals and π-π stacking interactions. The aromatic rings of compound **2** are oriented toward LEU361, MET447 and TYR472, contributing to a compact and stable fit without causing steric clashes. The centroid of compound **2** lies deep within hydrophobic groove, indicating its ability to penetrate effectively into the core of the binding pocket. In addition, compound **2** forms close-range hydrogen bond and π-π stacking interactions with Asp163 (3.14 Å) and Tyr472 (4.46 Å), both within favourable distance thresholds for stable binding. Furthermore, the hydrophobic tail is stabilised by its proximity to Leu361 (3.52 Å) and Met447(5.44 Å), which contributes to additional anchoring and overall complex stability. These close-range, non-covalent interactions, though individually weak, collectively contribute significantly to binding stability and specificity (Ferreira *et al*. 2015; Tsai *et al*. 2001).

In comparison, compound **1** binds within the catalytic pocket of *β*-glucuronidase in a slightly more superficial orientation than compound **2**. It forms several key hydrogen bonds with residues such as Lys568 (2.99 Å), Asn566 (2.99 Å), and Glu504 (2.28 Å), as well as π interactions with Tyr472 (4.02 Å) and Leu361 (5.34 Å). These interactions indicate that compound **1** has good spatial complementarity with the active site and contribute to the stability of the complex. Despite its shallower binding depth, it maintains favourable hydrophilic and hydrophobic interactions across the pocket. Its orientation also avoids steric clashes, allowing effective aromatic stacking and hydrogen bonding, which together support an energetically stable docking conformation.

Moving on to the control drug, DSA formed hydrogen bonds with key residues such as Tyr472, Asn566, Glu413, and Asp163, indicating moderate binding affinity. However, the presence of unfavourable donor–donor interactions with Lys568 and Arg562 suggests possible electrostatic repulsion or minor steric strain within the binding pocket. In the 3D structure, DSA appears to adopt a surface-exposed orientation, interacting near the edge of the catalytic site rather than being deeply embedded. This shallow and suboptimal binding may contribute to DSA’s weaker docking score and reduced inhibitory activity compared to compound **2**. A ligand with unsuitable geometry may face steric hindrance or poor alignment of interacting groups, both of which can significantly reduce binding affinity (Meng *et al*. 2011).

In general, docking studies in relation to SARs studies show that the methylenedioxyphenyl and carboxylic acid moieties in compound **2** contribute to binding affinity by forming the favourable interactions with amino acid within the active site. Furthermore, the molecular geometry of compound **2** plays a crucial role in contributing to the strength and stability of ligand-protein complex that influenced the binding affinity. Figure 4 shows compounds **1**, **2** and DSA aligned in the binding pocket of *β*-glucuronidase protein in 3D structures. Proper orientation of functional groups such as hydrogen bond donors and acceptors, hydrophobic moieties, or aromatic rings is essential for establishing key interactions, including hydrogen bonds, van der Waals forces, π-π stacking, and electrostatic interactions (Klebe *et al*. 2006; Kitchen *et al*. 2004).

## CONCLUSION

This is the first report on the *β*-glucuronidase inhibitory activity of endiandric acid isolated from *E. kingiana* bark. Based on comparative analysis of molecular docking and *in vitro* inhibitory assays, a consistent relationship was observed between predicted binding affinities and experimentally determined IC_50_ values for tsangibeilin B (**2**), kingianic acid A (**1**) and the positive control. Tsangibeilin B (**2**) showed the strongest docking score (−9.9 ± 0.1 kcal/mol^−1^) and demonstrated the most potent inhibitory activity *in vitro*, with an IC_50_ value of 7.5 ± 2.77 μM. This alignment supports the validity of the docking predictions for tsangibeilin B (**2**) and indicates a favourable fit within the *β*-glucuronidase active site, likely driven by strong hydrogen bonding and hydrophobic contacts that result in effective enzymatic inhibition. Thus, this study suggests that tsangibeilin B (**2**) could become a potent inhibitor and holds great promise as a natural *β*-glucuronidase inhibitor therapy. In future studies, these lead molecules should be further investigated for inhibition kinetic analysis and *in-vivo* anti-*β*-glucuronidase activities in appropriate animal models. In addition, the use of *E. coli β*-glucuronidase in this study serves as a preliminary screening model, therefore, further evaluation using mammalian *β*-glucuronidase is necessary to confirm physiological relevance.

## Figures and Tables

**FIGURE 1:**
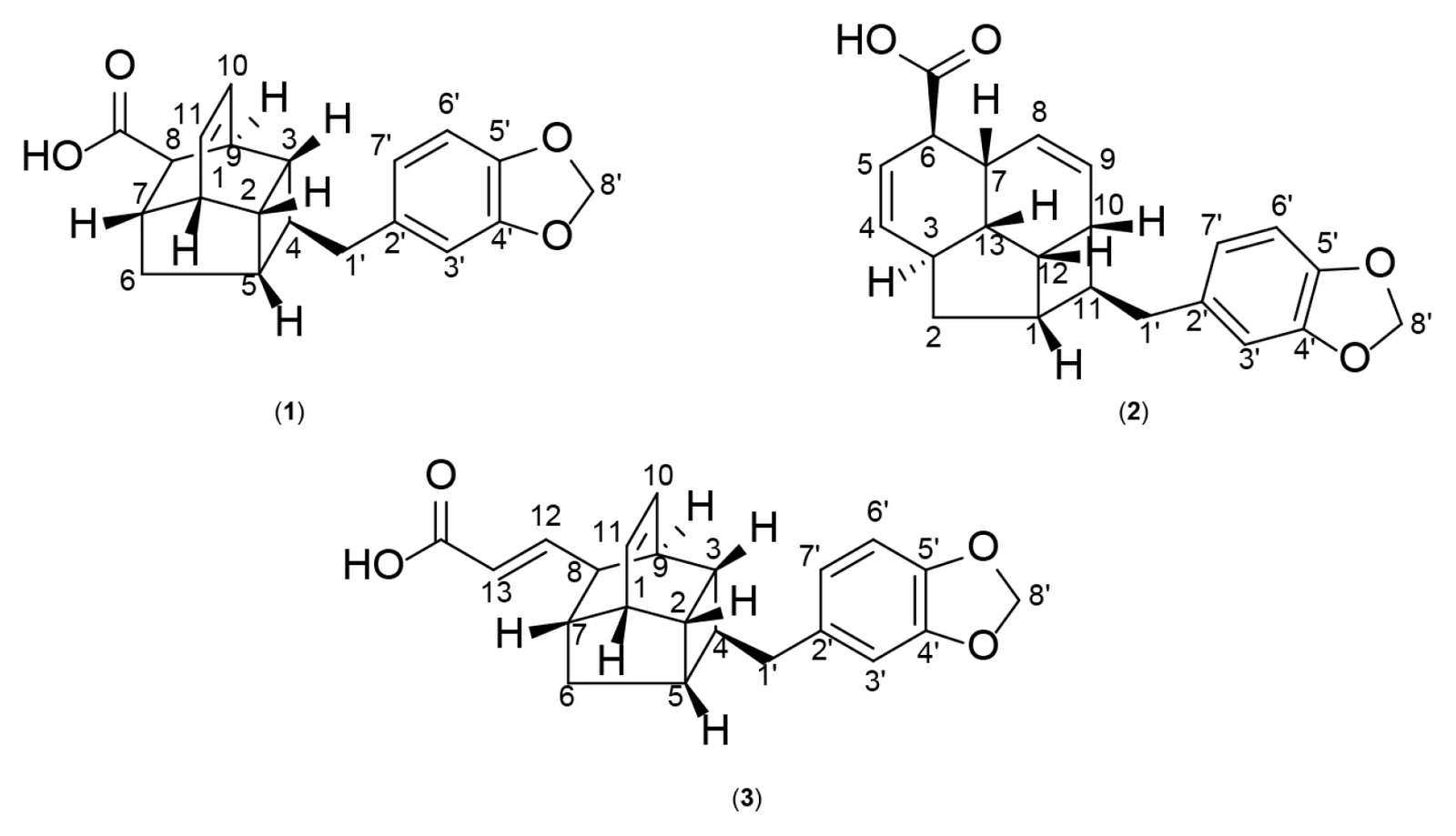
Chemical structure of the isolated compounds **1**–**3** from the bark of *E. Kingiana*.

**FIGURE 2:**
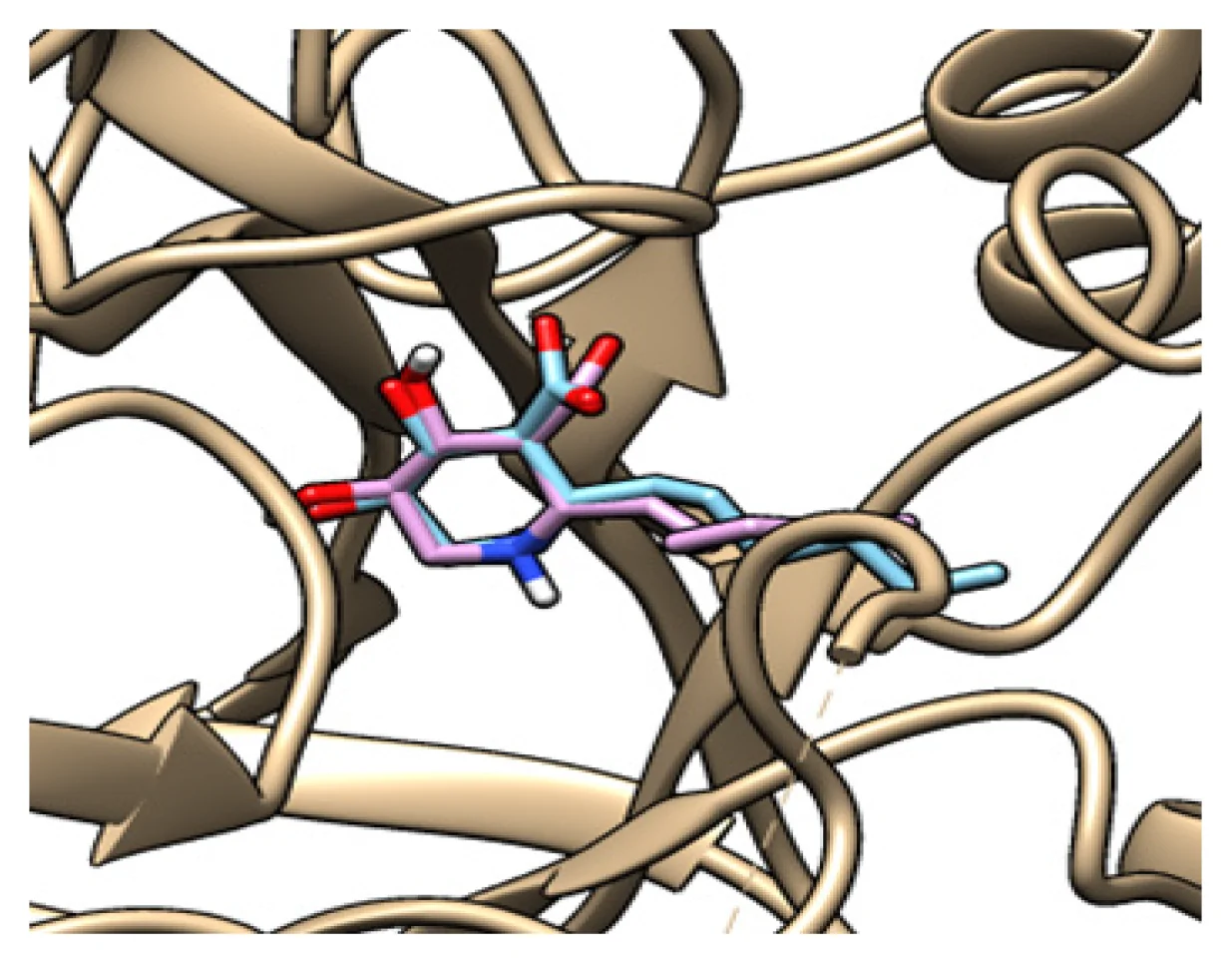
The superimposed co-crystallized ligand in purple (C6-hexyl uronic isofagomine) with extracted C6-hexyl uronic isofagomine (blue colour) redocked to *β*-glucuronidase crystal structure (PDB ID:6LEL).

**FIGURE 3:**
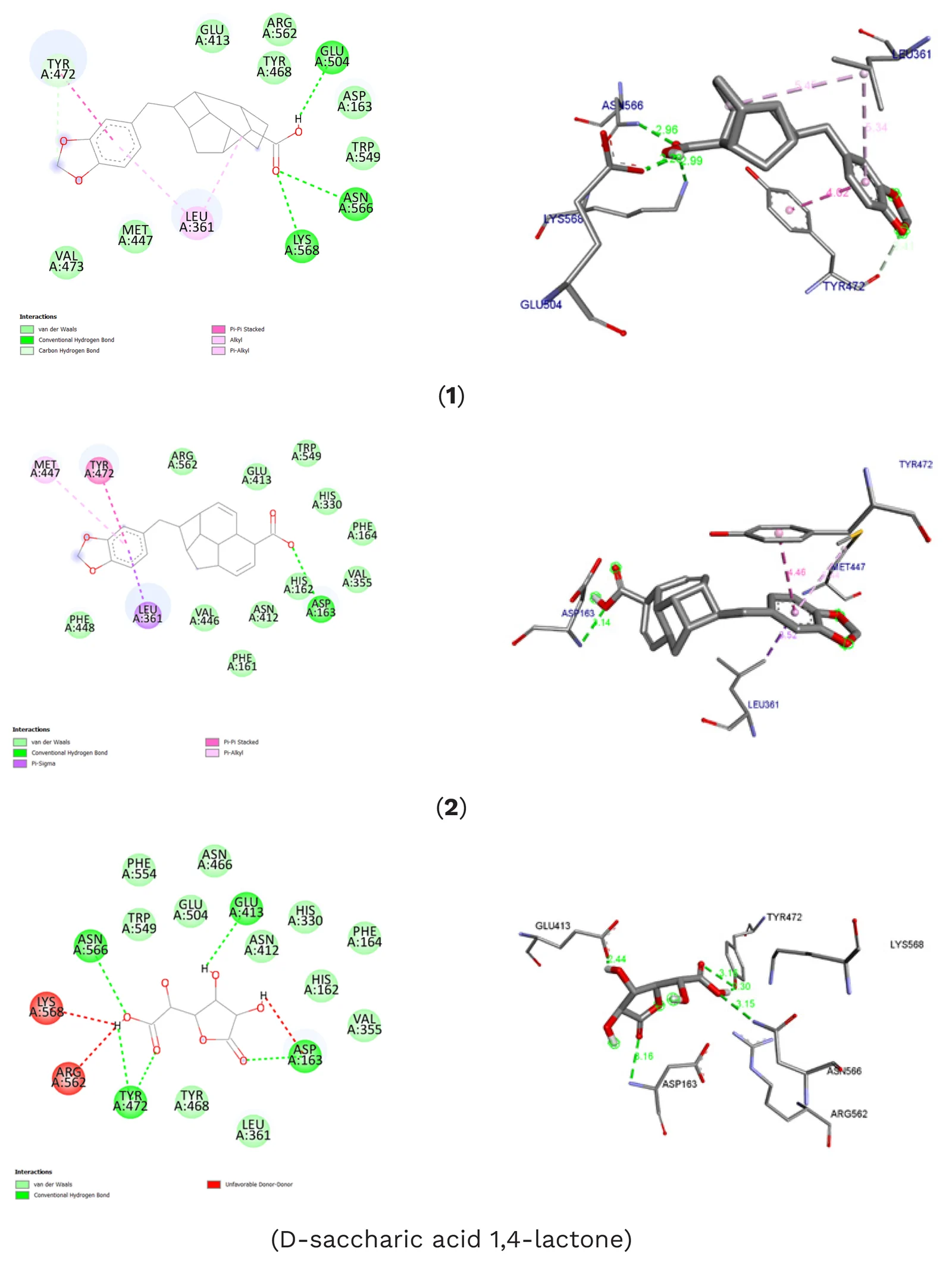
Illustration of 2D and 3D binding interactions of compound **1**, **2** and *D*-saccharic acid 1,4-lactone with *β*-glucuronidase protein.

**FIGURE 4:**
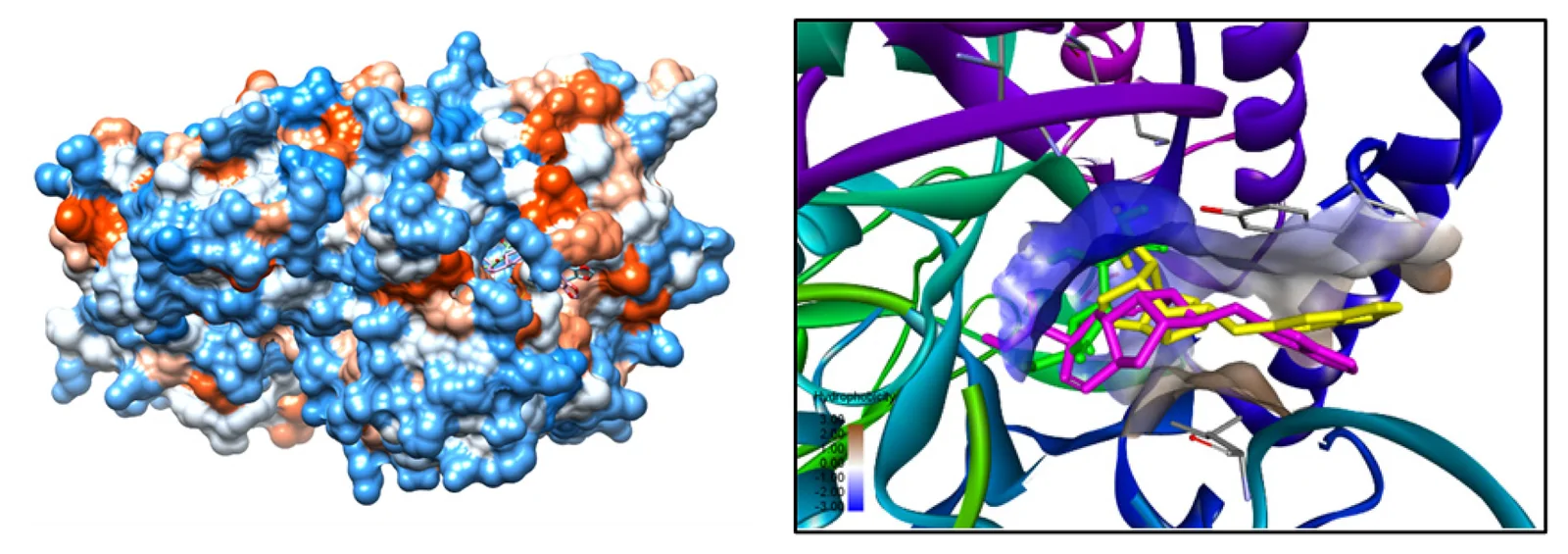
Compound **1** (yellow), **2** (pink) and *D*-saccharic acid 1,4-lactone (green) aligned in the binding pocket of *β*-glucuronidase protein in 3D structures.

**TABLE 1:** Activity of the compounds against *E. Coli β*-glucuronidase enzyme.

Compound	IC_50_ (μM)*
1	292 ± 5.40
2	7.5 ± 2.77
3	NA
D-saccharic acid 1,4-lactone	45.75 ± 2.16

*Notes*:

* Data reported as the mean ± standard deviation (SD) for n=3 independent experiments.

NA: Sample not available.

**TABLE 2:** *In silico* binding energy of tested compounds with *β*-glucuronidase protein (PDB id: 6LEL).

Protein	Compound	Binding energy, ΔG (kcal/mol^−1^)*
*β*-glucuronidase (PDB ID: 6LEL)	1	−9.3 ± 0.1
	2	−9.9 ± 0.1
	D-saccharic acid1,4-lactone	−7.0 ± 0.1

*Notes*:

* Mean ± standard deviation from at least three independent molecular docking.

**TABLE 3:** Binding interactions between isolated compounds and standard inhibitor with *β*-glucuronidase protein.

Compound	Bond	Protein interaction	Type of interaction	Distance (Å)
**1**	OH carboxylic acid	GLU504	Conventional hydrogen bond	2.49
	C=O carboxylic acid	LYS568	Conventional hydrogen bond	2.99
		ASN566	Conventional hydrogen bond	2.94
	Methylenedioxyphenyl	TYR472	Carbon Hydrogen bond	3.40
		TYR472	Pi-pi stacked	4.01
		LEU361	Pi-alkyl	5.37
	Cyclohexane	LEU361	Alkyl	5.47
**2**	OH carboxylic acid	ASP163	Conventional hydrogen bond	3.14
	Methylenedioxyphenyl	MET447	Pi-alkyl	5.44
		TYR472	Pi-pi stacked	4.46
		LEU361	Pi-sigma	3.52
*D*-saccharic acid 1,4-lactone	Hydroxyl	GLU413	Conventional hydrogen bond	2.44
	C=O carboxylic acid	TYR472	Conventional hydrogen bond	3.16
	OH carboxylic acid	TYR472	Conventional hydrogen bond	2.30
		ASN566	Conventional hydrogen bond	3.15
	C=O lactone ring	ASP163	Conventional hydrogen bond	3.16
